# The genome sequence of the Common Carder Bee, *Bombus pascuorum* (Scopoli, 1763)

**DOI:** 10.12688/wellcomeopenres.19251.1

**Published:** 2023-03-28

**Authors:** Liam M. Crowley, Olga Sivell, Duncan Sivell, University of Oxford and Wytham Woods Genome Acquisition Lab, Natural History Museum Genome Acquisition Lab, Darwin Tree of Life Barcoding collective, Wellcome Sanger Institute Tree of Life programme, Wellcome Sanger Institute Scientific Operations: DNA Pipelines collective, Tree of Life Core Informatics collective, Darwin Tree of Life Consortium

**Affiliations:** 1 University of Oxford, Oxford, England, UK; 2 Natural History Museum, London, England, UK

**Keywords:** Bombus pascuorum, Common Carder Bee, genome sequence, chromosomal, Hymenoptera

## Abstract

We present a genome assembly from an individual female *Bombus pascuorum* (the Common Carder Bee; Arthropoda; Insecta; Hymenoptera; Apidae). The genome sequence is 307.5 megabases in span. Most of the assembly is scaffolded into 17 chromosomal pseudomolecules. The mitochondrial genome has also been assembled and is 21.9 kilobases in length. Gene annotation of this assembly on Ensembl identified 12,999 protein coding genes.

## Species taxonomy

Eukaryota; Metazoa; Ecdysozoa; Arthropoda; Hexapoda; Insecta; Pterygota; Neoptera; Endopterygota; Hymenoptera; Apocrita; Aculeata; Apoidea; Apidae; *Bombus*; *Thoracobombus*; *Bombus pascuorum* (Scopoli, 1763) (NCBI:txid65598).

## Background

The Common Carder Bee, *Bombus pascuorum*, is one of the seven most common and widespread species of bumblebees in the UK and the only common and widespread species of the four all-brown carder bees. It has appeared to have replaced other carder bee species in northern Britain across recent decades ( Plowright *et al.*, 1997; Plowright & Plowright, 2009). It has a Palearctic distribution, with a range that includes all but the most northerly areas of western Europe through to China. It is found across a wide range of habitats, including gardens, grassland and woodlands.

It visits a wide range of flowers for nectar, especially species with longer corollae such as Fabaceae and Scrophulariacae ( Edwards & Jenner, 2005), being able to outcompete shorter-tongued bee species ( Balfour *et al.*, 2013). It is broadly polylectic, but shows a preference for pollen from Fabaceae, Scrophulariacae, Lamiaceae and red-flowered Asteraceae ( Edwards & Jenner, 2005). It had been shown to be less consistent in floral choice than other species of bumblebee ( Raine & Chittka, 2005). The presence of mass floral resources within the landscape may benefit this species by allowing increased colony sizes ( Herrmann *et al.*, 2007). In common with other species in the genus, the high degree of floral visitation undertaken by this species indicates its important role as a pollinator.

*Bombus pascuorum* is a eusocial species with reproductive queens and males, and non-reproductive workers. Queens, workers and males are all buff/brown all over, although it is highly variable in appearance with differing degrees of dark and pale hairs. It can be distinguished from other all-brown bumblebee species in the UK by the presence of at least some black hairs on the abdomen.

It has an exceptionally long flight period, with queens emerging from winter diapause from March, the first workers from April and reproductives from July. It is possibly bivoltine in the UK, often being the latest flying bumblebee species that does not remain active throughout the winter, with workers present into October.

Nests are constructed above ground, typically in grass tussocks, under plant litter or at the base of shrubs and trees. It is one of four carder bee species, referring to the construction of a moss and dry grass covering to the nest. Fewer workers are produced than other bumblebee species, with nests peaking between 60 and 150 workers ( Løken, 1973; von Hagen, 2003). In UK agricultural landscapes, nest density is estimated at 68 nests per km ^2^, and minimum estimated maximum foraging range 450 m ( Knight *et al.*, 2005).

A complete genome sequence for this species will facilitate studies into the evolution of eusociality, conservation of important pollinator species, reproductive evolution and foraging behaviour.

## Genome sequence report

The genome was sequenced from one female *Bombus pascuorum* specimen collected from Wytham Woods, Oxfordshire, UK (latitude 51.77, longitude –1.34). A total of 28-fold coverage in Pacific Biosciences single-molecule HiFi long reads and 92-fold coverage in 10X Genomics read clouds were generated. Primary assembly contigs were scaffolded with chromosome conformation Hi-C data. Manual assembly curation corrected 36 missing joins or mis-joins and removed three haplotypic duplications, reducing the assembly length by 0.98%% and the scaffold number by 21.36%, and increasing the scaffold N50 by 45.58%.

**Figure 1.**
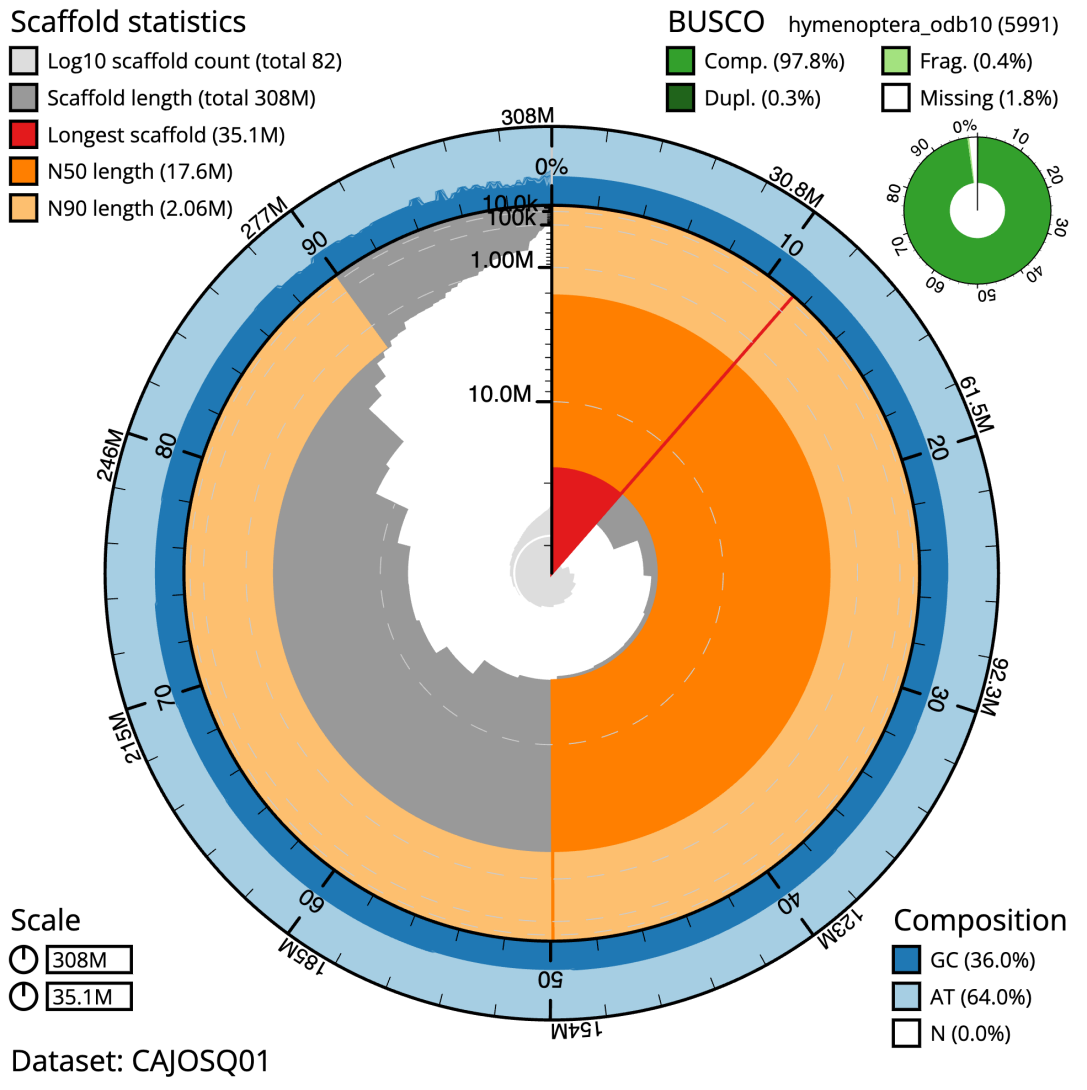
Genome assembly of *Bombus pascuorum*, iyBomPasc1.1: metrics. The BlobToolKit Snailplot shows N50 metrics and BUSCO gene completeness. The main plot is divided into 1,000 size-ordered bins around the circumference with each bin representing 0.1% of the 307,570,237 bp assembly. The distribution of scaffold lengths is shown in dark grey with the plot radius scaled to the longest scaffold present in the assembly (35,072,465 bp, shown in red). Orange and pale-orange arcs show the N50 and N90 scaffold lengths (17,556,731 and 2,056,671 bp), respectively. The pale grey spiral shows the cumulative scaffold count on a log scale with white scale lines showing successive orders of magnitude. The blue and pale-blue area around the outside of the plot shows the distribution of GC, AT and N percentages in the same bins as the inner plot. A summary of complete, fragmented, duplicated and missing BUSCO genes in the hymenoptera_odb10 set is shown in the top right. An interactive version of this figure is available at https://blobtoolkit.genomehubs.org/view/iyBomPasc1.1/dataset/CAJOSQ01/snail.

The final assembly has a total length of 307.5 Mb in 81 sequence scaffolds with a scaffold N50 of 17.6 Mb ( Table 1). Most (87.82%) of the assembly sequence was assigned to 17 chromosomal-level scaffolds. Chromosome-scale scaffolds confirmed by the Hi-C data are named in order of size ( Figure 1– Figure 4; Table 2). While not fully phased, the assembly deposited is of one haplotype. Contigs corresponding to the second haplotype have also been deposited.

**Table 1.** Genome data for *Bombus pascuorum*, iyBomPasc1.1.

Project accession data		
Assembly identifier	iyBomPasc1.1	
Species	*Bombus pascuorum*	
Specimen	iyBomPasc1	
NCBI taxonomy ID	65598	
BioProject	PRJEB43540	
BioSample ID	SAMEA7520484	
Isolate information	iyBomPasc1, male, head and thorax (DNA sequencing and Hi-C scaffolding) iyBomPasc2, thorax (RNA sequencing)	
Assembly metrics *		*Benchmark*
Consensus quality (QV)	51.4	*≥ 50*
*k*-mer completeness	99.98%	*≥ 95%*
BUSCO **	C:97.8%[S:97.5%,D:0.3%], F:0.4%,M:1.8%,n:5,991	*C ≥ 95%*
Percentage of assembly mapped to chromosomes	87.82%	*≥ 95%*
Sex chromosomes	Not applicable	*localised homologous pairs*
Organelles	Mitochondrial genome assembled	*complete single alleles*
**Raw data accessions**		
PacificBiosciences SEQUEL II	ERR6548408	
10X Genomics Illumina	ERR6054545–ERR6054548	
Hi-C Illumina	ERR6054549, ERR6054550	
PolyA RNA-Seq Illumina	ERR6054551, ERR6286716	
Genome assembly		
Assembly accession	GCA_905332965.1	
*Accession of alternate haplotype*	GCA_905332995.1	
Span (Mb)	307.5	
Number of contigs	120	
Contig N50 length (Mb)	9.0	
Number of scaffolds	81	
Scaffold N50 length (Mb)	17.6	
Longest scaffold (Mb)	35.1	
Genome annotation		
Number of protein-coding genes	12,999	
Number of non-protein coding genes	5,442	
Average length of coding sequence (bp)	12,765.73	
Average number of exons per transcript	6.13	
Average number of introns per transcript	5.13	

^*^Assembly metric benchmarks are adapted from column VGP-2020 of “Table 1: Proposed standards and metrics for defining genome assembly quality” from ( Rhie *et al.*, 2021). ^**^BUSCO scores based on the hymenoptera_odb10 BUSCO set using v5.3.2. C = complete [S = single copy, D = duplicated], F = fragmented, M = missing, n = number of orthologues in comparison. A full set of BUSCO scores is available at https://blobtoolkit.genomehubs.org/view/iyBomPasc1.1/dataset/CAJOSQ01/busco.

The estimated Quality Value (QV) of the final assembly is 51.4 with *k*-mer completeness of 99.98%, and the assembly has a BUSCO v5.3.2 ( Manni *et al*., 2021) completeness of 97.8% (single 97.5%, duplicated 0.3%) using the hymenoptera_odb10 reference set ( *n* = 5,991).

**Figure 2.**
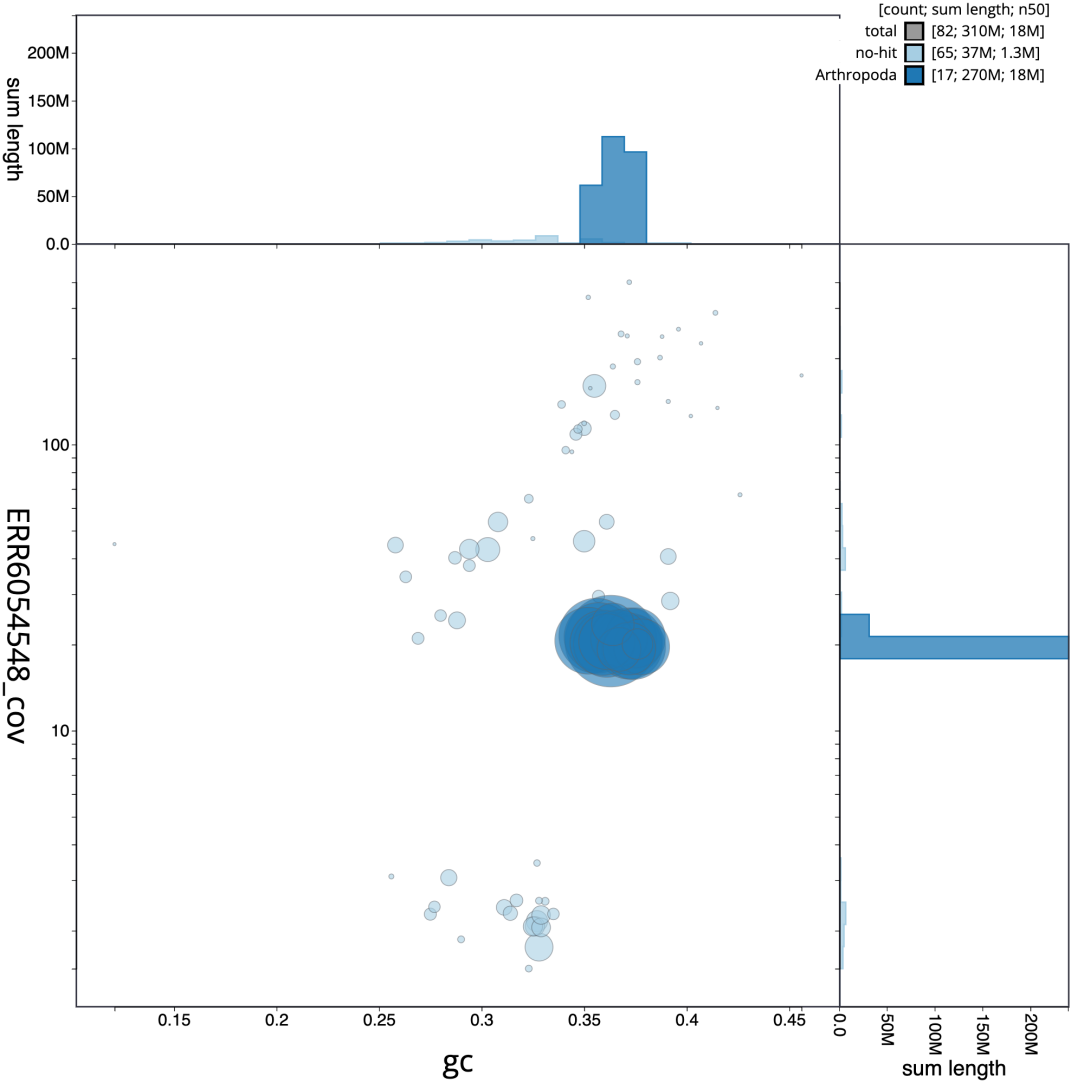
Genome assembly of *Bombus pascuorum*, iyBomPasc1.1: GC coverage. BlobToolKit GC-coverage plot. Scaffolds are coloured by phylum. Circles are sized in proportion to scaffold length. Histograms show the distribution of scaffold length sum along each axis. An interactive version of this figure is available at https://blobtoolkit.genomehubs.org/view/iyBomPasc1.1/dataset/CAJOSQ01/blob.

**Figure 3.**
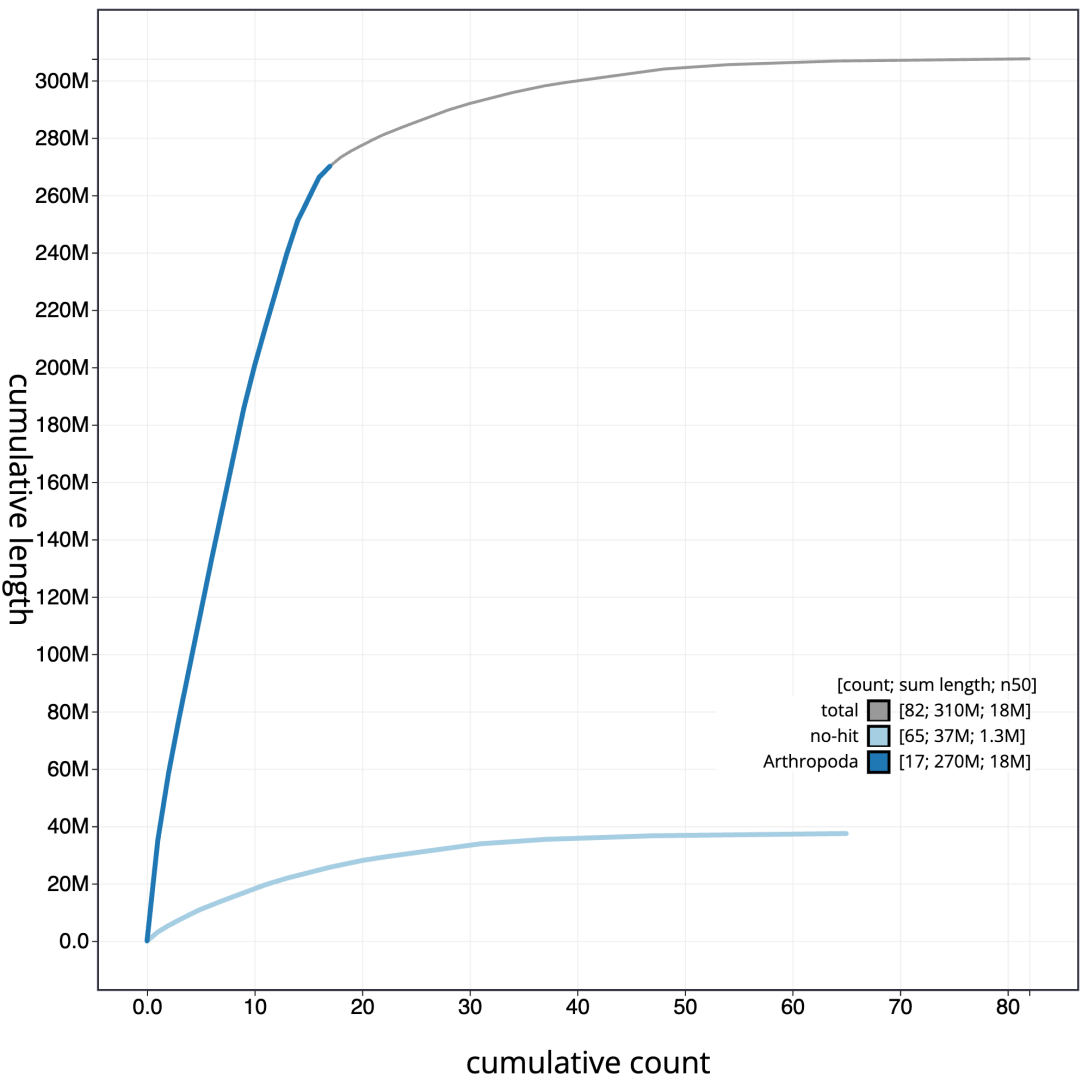
Genome assembly of *Bombus pascuorum*, iyBomPasc1.1: cumulative sequence. BlobToolKit cumulative sequence plot. The grey line shows cumulative length for all scaffolds. Coloured lines show cumulative lengths of scaffolds assigned to each phylum using the buscogenes taxrule. An interactive version of this figure is available at https://blobtoolkit.genomehubs.org/view/iyBomPasc1.1/dataset/CAJOSQ01/cumulative.

**Figure 4.**
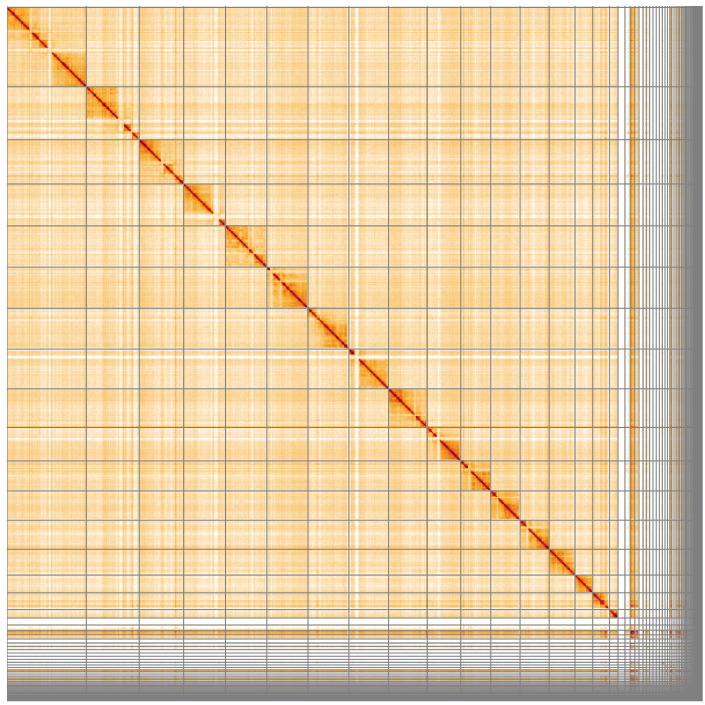
Genome assembly of *Bombus pascuorum*, iyBomPasc1.1: Hi-C contact map. Hi-C contact map of the iyBomPasc1.1 assembly, visualised using HiGlass. Chromosomes are shown in order of size from left to right and top to bottom. An interactive version of this figure may be viewed at https://genome-note-higlass.tol.sanger.ac.uk/l/?d=I2eUKHqSTa-R7bZ0rOEWwg.

**Table 2.** Chromosomal pseudomolecules in the genome assembly of *Bombus pascuorum*, iyBomPasc1.

INSDC accession	Chromosome	Size (Mb)	GC%
HG995268.1	1	35.07	36.3
HG995269.1	2	23.41	35.6
HG995270.1	3	19.61	35.7
HG995271.1	4	18.51	35.2
HG995272.1	5	18.27	36.1
HG995273.1	6	18.2	37.2
HG995274.1	7	18.05	37.2
HG995275.1	8	17.56	35.9
HG995276.1	9	17.12	37.4
HG995277.1	10	14.79	37.5
HG995278.1	11	13.28	36.7
HG995279.1	12	12.94	37.8
HG995280.1	13	12.9	36.1
HG995281.1	14	11.4	37.2
HG995282.1	15	7.83	36.7
HG995283.1	16	7.38	36.4
HG995284.1	17	3.78	37.6
HG995285.1	MT	0.02	12.1
-	unplaced	37.44	32.4

## Genome annotation report

The *B. pascuorum* genome assembly GCA_905332965.1 was annotated using the Ensembl rapid annotation pipeline ( Table 1; https://rapid.ensembl.org/Bombus_pascuorum_GCA_905332965.1/Info/Index/). The resulting annotation includes 12,999 protein coding genes with an average length of 12,765.73 and an average coding length of 1,417.90, and 5,443 non-protein coding genes. There is an average of 6.13 exons and 5.13 introns per canonical protein coding transcript, with an average intron length of 1646.99. A total of 7659 gene loci have more than one associated transcript.

## Methods

### Sample acquisition and nucleic acid extraction

A female *B. pascuorum* specimen (iyBomPasc1) was collected from Wytham Woods, Oxfordshire (biological vice-county: Berkshire), UK (latitude 51.77, longitude –1.34) on 7 August 2019. The specimen was taken from woodland by Liam Crowley (University of Oxford) by netting. The specimen was identified by the collector and snap-frozen on dry ice. This specimen was used for genome sequencing and Hi-C scaffolding.

A second female *B. pascuorum* specimen (iyBomPasc2) was used for RNA sequencing. The iyBomPasc2 specimen was collected by Olga Sivell (Natural History Museum) from woodland edge in Luton, UK (latitude 51.88, longitude –0.37) on 5 May 2020. The specimen was identified by Duncan Sivell (Natural History Museum) and snap-frozen on dry ice.

DNA was extracted at the Tree of Life laboratory, Wellcome Sanger Institute (WSI). The iyBomPasc1 sample was weighed and dissected on dry ice with tissue set aside for Hi-C sequencing. Head and thorax tissue was disrupted using a Nippi Powermasher fitted with a BioMasher pestle. High molecular weight (HMW) DNA was extracted using the Qiagen MagAttract HMW DNA extraction kit. Low molecular weight DNA was removed from a 20 ng aliquot of extracted DNA using the 0.8X AMpure XP purification kit prior to 10X Chromium sequencing; a minimum of 50 ng DNA was submitted for 10X sequencing. HMW DNA was sheared into an average fragment size of 12–20 kb in a Megaruptor 3 system with speed setting 30. Sheared DNA was purified by solid-phase reversible immobilisation using AMPure PB beads with a 1.8X ratio of beads to sample to remove the shorter fragments and concentrate the DNA sample. The concentration of the sheared and purified DNA was assessed using a Nanodrop spectrophotometer and Qubit Fluorometer and Qubit dsDNA High Sensitivity Assay kit. Fragment size distribution was evaluated by running the sample on the FemtoPulse system.

RNA was extracted from thorax tissue of iyBomPasc2 in the Tree of Life Laboratory at the WSI using TRIzol, according to the manufacturer’s instructions. RNA was then eluted in 50 μl RNAse-free water and its concentration assessed using a Nanodrop spectrophotometer and Qubit Fluorometer using the Qubit RNA Broad-Range (BR) Assay kit. Analysis of the integrity of the RNA was done using Agilent RNA 6000 Pico Kit and Eukaryotic Total RNA assay.

### Sequencing

Pacific Biosciences HiFi circular consensus and 10X Genomics read cloud DNA sequencing libraries were constructed according to the manufacturers’ instructions. Poly(A) RNA-Seq libraries were constructed using the NEB Ultra II RNA Library Prep kit. DNA and RNA sequencing were performed by the Scientific Operations core at the WSI on Pacific Biosciences SEQUEL II (HiFi), Illumina HiSeq 4000 (RNA-Seq) and HiSeq X Ten (10X) instruments. Hi-C data were also generated from tissue of iyBomPasc1 using the Arima v2 kit and sequenced on the HiSeq X Ten instrument.

### Genome assembly, curation and evaluation

Assembly was carried out with Hifiasm ( Cheng *et al.*, 2021) and haplotypic duplication was identified and removed with purge_dups ( Guan *et al.*, 2020). One round of polishing was performed by aligning 10X Genomics read data to the assembly with Long Ranger ALIGN, calling variants with FreeBayes ( Garrison & Marth, 2012). The assembly was then scaffolded with Hi-C data ( Rao *et al.*, 2014) using SALSA2 ( Ghurye *et al.*, 2019). The assembly was checked for contamination and corrected using the gEVAL system ( Chow *et al.*, 2016) as described previously ( Howe *et al.*, 2021). Manual curation was performed using gEVAL, HiGlass ( Kerpedjiev *et al.*, 2018) and Pretext ( Harry, 2022). The mitochondrial genome was assembled using MitoHiFi ( Uliano-Silva *et al.*, 2022), which performed annotation using MitoFinder ( Allio *et al.*, 2020). To evaluate the assembly, MerquryFK was used to estimate consensus quality (QV) scores and *k*-mer completeness ( Rhie *et al.*, 2020). The genome was analysed and BUSCO scores ( Manni *et al.*, 2021; Simão *et al.*, 2015) were calculated within the BlobToolKit environment ( Challis *et al.*, 2020). Table 3 contains a list of software tool versions and sources.

**Table 3.** Software tools: versions and sources.

Software tool	Version	Source
BlobToolKit	4.0.7	https://github.com/blobtoolkit/blobtoolkit
BUSCO	5.3.2	https://gitlab.com/ezlab/busco
FreeBayes	1.3.1-17-gaa2ace8	https://github.com/freebayes/freebayes
gEVAL	N/A	https://geval.org.uk/
Hifiasm	0.12	https://github.com/chhylp123/hifiasm
HiGlass	1.11.6	https://github.com/higlass/higlass
Long Ranger ALIGN	2.2.2	https://support.10xgenomics.com/genome-exome/ software/pipelines/latest/advanced/other-pipelines
Merqury	MerquryFK	https://github.com/thegenemyers/MERQURY.FK
MitoHiFi	1	https://github.com/marcelauliano/MitoHiFi
PretextView	0.2	https://github.com/wtsi-hpag/PretextView
purge_dups	1.2.3	https://github.com/dfguan/purge_dups
SALSA	2.2	https://github.com/salsa-rs/salsa

### Genome annotation

The Ensembl gene annotation system ( Aken *et al.*, 2016) was used to generate annotation for the *Bombus pascuorum* assembly (GCA_905332965.1). Annotation was created primarily through alignment of transcriptomic data to the genome, with gap filling via protein-to-genome alignments of a select set of proteins from UniProt ( UniProt Consortium, 2019).

### Ethics and compliance issues

The materials that have contributed to this genome note have been supplied by a Darwin Tree of Life Partner. The submission of materials by a Darwin Tree of Life Partner is subject to the Darwin Tree of Life Project Sampling Code of Practice. By agreeing with and signing up to the Sampling Code of Practice, the Darwin Tree of Life Partner agrees they will meet the legal and ethical requirements and standards set out within this document in respect of all samples acquired for, and supplied to, the Darwin Tree of Life Project. All efforts are undertaken to minimise the suffering of animals used for sequencing. Each transfer of samples is further undertaken according to a Research Collaboration Agreement or Material Transfer Agreement entered into by the Darwin Tree of Life Partner, Genome Research Limited (operating as the Wellcome Sanger Institute), and in some circumstances other Darwin Tree of Life collaborators.

## Data Availability

European Nucleotide Archive: B *ombus pascuorum* (common carder bee). Accession number PRJEB43540; https://identifiers.org/ena.embl/PRJEB43540. ( Wellcome Sanger Institute, 2021) The genome sequence is released openly for reuse. The *Bombus pascuorum* genome sequencing initiative is part of the Darwin Tree of Life (DToL) project. All raw sequence data and the assembly have been deposited in INSDC databases. Raw data and assembly accession identifiers are reported in Table 1.

## Author information

Members of the University of Oxford and Wytham Woods Genome Acquisition Lab are listed here: https://doi.org/10.5281/zenodo.4789928.

Members of the Darwin Tree of Life Barcoding collective are listed here: https://doi.org/10.5281/zenodo.4893703.

Members of the Wellcome Sanger Institute Tree of Life programme are listed here: https://doi.org/10.5281/zenodo.4783585.

Members of Wellcome Sanger Institute Scientific Operations: DNA Pipelines collective are listed here: https://doi.org/10.5281/zenodo.4790455.

Members of the Tree of Life Core Informatics collective are listed here: https://doi.org/10.5281/zenodo.5013541.

Members of the Darwin Tree of Life Consortium are listed here: https://doi.org/10.5281/zenodo.4783558.
