# Supplementary material for: The genome sequence of the Common Carder Bee, Bombus pascuorum (Scopoli, 1763)
Source: Wellcome Open Res. 2023 Mar 28;8:142. [Version 1] doi: 10.12688/wellcomeopenres.19251.1 (PMC10444996; doi:10.12688/wellcomeopenres.19251.1)
Supplement: Peer review history

# Peer review: Reviewer response for version 1

**DOI:** 10.21956/wellcomeopenres.21334.r63871

**Published:** 2023-08-22

**Authors:** Rodolpho S. T. Menezes

**Affiliations:** 1 Department of Biological Sciences, State University of Santa Cruz, Ilhéus, Brazil

This genome report describes the genome sequencing of the common carder bee, *Bombus pascuorum*. This genome will be interesting for comparative genomics investigation within Apidae and also for evolution and conservation studies. Moreover, the manuscript is well written and therefore it can be published in this journal. Below I list few comments to the authors to improve the manuscript:

- Genome assembly: Since you assembled mitochondrial genome I think it is interesting you show general (GC/AT content…) mitochondrial information about this species.
- Table 3: Please include MitoFinder information.

Are sufficient details of methods and materials provided to allow replication by others?

Yes

Is the rationale for creating the dataset(s) clearly described?

Yes

Are the datasets clearly presented in a useable and accessible format?

Yes

Are the protocols appropriate and is the work technically sound?

Yes

Reviewer Expertise:

Cytogenetics, Biogeography, Phylogenomics.

I confirm that I have read this submission and believe that I have an appropriate level of expertise to confirm that it is of an acceptable scientific standard.

# Peer review: Reviewer response for version 1

**DOI:** 10.21956/wellcomeopenres.21334.r63874

**Published:** 2023-08-22

**Authors:** Peter K. Dearden

**Affiliations:** 1 University of Otago, Dunedin, Otago, New Zealand

This manuscript presents the genome of the common carder bee, a eusocial, haplodiploid bumble bee species widely distributed in Europe.

 The genome was assembled from a single female bee, and RNAseq used to annotate genes form a second specimen. Given the species is haplodiploid, it would be interesting to know why the authors didn't use a male as this would have produced a fully phased assembly.

 The tools and methods used are entirely appropriate for this assembly, and the genome produced is of excellent quality and will be a key resource for bumble bee and insect researchers.

Are sufficient details of methods and materials provided to allow replication by others?

Yes

Is the rationale for creating the dataset(s) clearly described?

Yes

Are the datasets clearly presented in a useable and accessible format?

Yes

Are the protocols appropriate and is the work technically sound?

Yes

Reviewer Expertise:

insect genomics

I confirm that I have read this submission and believe that I have an appropriate level of expertise to confirm that it is of an acceptable scientific standard.
